# Identifying specific matrix metalloproteinase-2-inhibiting peptides through phage display-based subtractive screening

**DOI:** 10.3906/biy-2105-6

**Published:** 2021-12-14

**Authors:** Aylin ÖZDEMİR BAHADIR, Bertan Koray BALCIOĞLU, Müge SERHATLI, Şeyma IŞIK, Berrin ERDAĞ

**Affiliations:** 1 Genetic Engineering and Biotechnology Institute, Marmara Research Center, TÜBİTAK, Kocaeli Turkey; 2 Department of Medical Biotechnology Institute of Health Sciences Acıbadem Mehmet Ali Aydınlar University, İstanbul Turkey; 3 Department of Medical Biology, Basic Medical Sciences, İstanbul Aydın University, İs-tanbul Turkey

**Keywords:** Phage display, peptide, matrix metalloproteinases, MMP-2, subtractive panning

## Abstract

Gelatinases A and B, which are members of the matrix metalloproteinase (MMP) family, play essential roles in cancer development and metastasis, as they can break down basal membranes. Therefore, the determination and inhibition of gelatinases is essential for cancer treatment. Peptides that can specifically block each gelatinase may, therefore, be useful for cancer treatment. In this study, subtractive panning was carried out using a 12-mer peptide library to identify peptides that block gelatinase A activity (MMP-2), which is a key pharmacological target. Using this method, 17 unique peptide sequences were determined. MMP-2 inhibition by these peptides was evaluated through zymogram analyses, which revealed that four peptides inhibited MMP-2 activity by at least 65%. These four peptides were synthesized and used for in vitro wound healing using human umbilical vein endothelial cells, and two peptides, AOMP12 and AOMP29, were found to inhibit wound healing by 40%. These peptides are, thus, potential candidates for MMP-2 inhibition for cancer treatment. Furthermore, our findings suggest that our substractive biopanning screening method is a suitable strategy for identifying peptides that selectively inhibit MMP-2.

## 1. Introduction

The extracellular matrix (ECM), also referred to as the connective tissue, is a complex structure that surrounds and supports cells in mammalian tissues. The remodeling of tissues and the regulation of cellular migration are linked to the controlled destruction of the ECM by a special group of endopeptidase enzymes. Matrix metalloproteinases (MMPs), collectively called matrixins, are involved in ECM degradation. The MMP family of enzymes consists of more than 20 types (Pilcher et al., 1999; Vihinen and Kähäri, 2002; Dufour and Overall, 2013). MMPs are secreted primarily from the mesenchyme and fibroblasts during tissue development and regeneration. Normal physiological events, such as embryonic development, inflammatory cell migration, wound healing, and angiogenesis are modulated by the activity of extracellular enzymes and natural inhibitors of MMPs. In particular, MMPs are expressed at low levels in normal tissue but are expressed at high levels in cases of inflammation and physiological remodeling alongside other biomarkers, such as cytokines, developmental factors, and ECM components. Disrupted expression and activation of MMPs have been linked to diseases such as cancer, autoimmune diseases, tissue ulcers, atherosclerosis, and aneurysm (Nagaset and Woessner, 1999).

Gelatinases A and B (MMP-2 and MMP-9, respectively) are the most important members of the MMP family. Unlike other MMPs, MMP-2 and MMP-9 break up gelatins, one of the main elements of the basal membrane. They are secreted as zymogens and then cleaved into the active form (Cieplak and Strongin, 2017). In metastatic tumors, type IV collagen activity is high as the metastatic cells need to replace the basal membrane and blood vessels. MMPs secreted by tumor cells, stromal fibroblasts, and infiltrating inflammatory cells play key roles in various stages of tumor cell invasion and metastatic progression (Deryugina and Quigley, 2006). MMP-2 and -9 are known to be essential to malignant cancer invasion by disrupting the surrounding ECM and accelerating cancer metastasis and angiogenesis (Giannopoulos et al., 2008; Kumar et al., 2010). Therefore, both gelatinases, primarily MMP-2, are good targets for anticancer drugs. MMPs also play a role in angiogenesis by disrupting the vascular basement membrane and by remodeling the ECM (Overall and López-Otín, 2002; Chakraborti et al., 2003). Therefore, these enzymes are important targets for the development of antitumor drugs. Furthermore, studies have shown that these enzymes may be used as markers for cancer diagnosis and monitoring cancer progression (Roy et al., 2009; Huang, 2018).

Inhibition of tumor metastasis and angiogenesis through the suppression of MMP activity is, thus, considered a promising strategy for cancer treatment (Cao, 2001; Guruvayoorappan and Kuttan, 2008; Raeeszadeh-Sarmazdeh M 2020). Currently, approximately 60 drug candidates targeting different MMPs have been developed for the treatment of cancer, cardiovascular diseases, and tissue inflammation. Except for Periostat, which has been approved for periodontitis, these candidates have not been successful due to side effects because of their lack of specificity (Levin et al., 2017). However, the development of chemicals that selectively inhibit specific MMPs has been challenging. One strategy to overcome the issues on specificity and toxicity of chemical MMP inhibitors is through the development of biological structures that are unique to the target MMP subtype. Therapeutic peptides are preferred owing to their high specificity, selectivity, and low toxicity (Marqus et al., 2017). 

The phage display technology is a powerful technique wherein unique protein or peptide structures can be selected through biopanning against a target. In this study, the phage display technique was used to determine 12-mer peptides that specifically bind active MMP-2. First, a peptide library was selected for binding against active MMP-9, and phages that bind active MMP-9 were eliminated. Then, the library was biopanned for interaction with active MMP-2. Subtractive biopanning was performed in this manner for a number of cycles to enrich for specific MMP-2-binding peptides. The selected peptides were identified and further analyzed for their gelatinase inhibitory capacities in the hopes of discovering peptide candidates for MMP-2 inhibition and, ultimately, for cancer treatment.

## 2. Materials and methods

### 2.1. Bacterial strains

*Escherichia coli* TG1 (*E. coli* TG1; supE, hsdΔ5, thiΔ(lac-proAB), F` [traD36 proAB+lacIq lacZΔM15]; Amersham Pharmacia Biotech, Buckinghamshire, United Kingdom) was used as the host for phage infection and production.

### 2.2. Peptide library

A rationally designed combinatorial phage display library (Ph.D.-12 Phage Display Peptide Library) of 12-mer peptide sequences was obtained from New England Biolabs or Fermentas, Inc., Beverly, MA. Each 12-mer peptide sequence was inserted into the NH_2_ terminus of the pIII minor coat protein of the M13 bacteriophage. The peptide sequence was followed by a short spacer (Gly-Gly-Gly-Ser) and the wild-type pIII sequence.

### 2.3. Gelatinase activation

The enzymes MMP-2 (Sigma, M1827) and MMP-9 (Sigma, M4809) were incubated and activated in 1 mM amino-phenyl mercuric acetate (APMA, Merck KGaA, Darmstadt, Germany) at 37 °C for 2 h (Koivunen et al., 1999). The enzymes were then subjected to sodium dodecyl sulfate-polyacrylamide gel electrophoresis (SDS-PAGE) and visualized through Coomassie staining (Sambrook et al., 1989).

### 2.4. Biopanning

**Figure 1:**
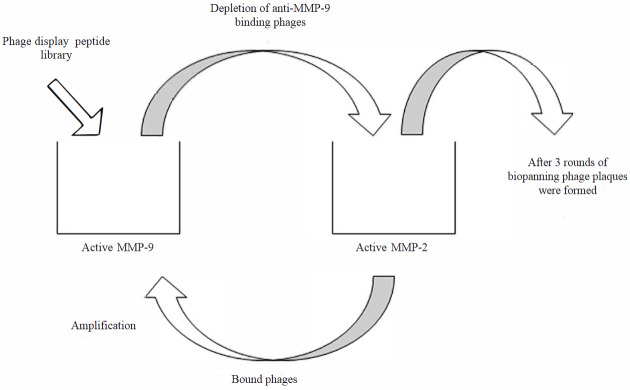
Schematic representation of the subtractive selection process on a phage display peptide library. The phage library was exposed to active matrix metalloproteinase (MMP)-9 to deplete MMP-9-binding phages. Phages that did not bind MMP-9 were exposed to active MMP-2. The bound pages were then eluted and amplified and exposed again to active MMP-9. This cycle was repeated four times. After the last cycle, the recovered phage clones were amplified, titrated, and sequenced.

The strategy used for one biopanning round of the Ph.D.-12 peptide library to select the MMP-2 specific peptide is shown in Figure 1. Wells of a high-binding microtiter plate (TPP, Trasadingen, Switzerland) were coated with 250 ng/200 μL of activated MMP-2 or MMP-9. The wells were washed three times with phosphate-buffered saline (PBS) containing 0.1% Tween 20 (TPBS), and the wells were blocked with 4% milk powder without fat for 2 h at 4 °C. For subtractive screening, 200 µL of the Ph.D.-12 library (containing 4 × 10^10^ phages) was first added to wells coated with active MMP-9 figand incubated for 1 h at room temperature. Unbound phages were recovered and transferred to wells coated with active-MMP-2. After 1 h of incubation, unbound phages were discarded by intensive washing (30 times TPBS and then 30 times with PBS). Phages bound to active-MMP2 were eluted with 200 µL elution buffer (0.2 M glycine-HCl, pH = 2.2) and amplified in E. coli TG1. The amplified phages were subjected to another three rounds of selective screening as aforementioned, to enrich for clones that are specific to active MMP-2. After a total of four rounds of biopanning, the MMP-2-specific clones were plated, and single pure plaques were isolated through phage amplification (Smith and Scott, 1993). 

### 2.5. Phage amplification

Overnight *E. coli* TG1 cultures were refreshed (1/100 volume) in 20 mL nutrient broth, and phage solutions were added to the cultures. The cultures were incubated for 4 h at 37 °C with shaking at 220 rpm. The culture was then centrifuged at 10.000 rpm for 10 min at 4 °C. The supernatant was then centrifuged again under the same conditions. Then, 80% of the supernatant was collected, and polyethylene glycol solution (1/6 of total volume; PEG8000 (Merck KGaA, Darmstadt, Germany)/ 2.5 M NaCl) was added. The solution was incubated on ice for 1 h. The solution was centrifuged again at 10,000 rpm for 15 min at 4 °C. The pellet was dissolved in 1 mL of Tris-buffered saline (TBS), 1/6 volume of PEG8000/ 2.5 M NaCl solution was added, and the resuspensions were incubated on ice for half an hour. Then, the mixture was centrifuged at 10,000 rpm for 15 min at 4 °C. The pellet containing the phages was dissolved in a TBS. The number of phages was determined as plaque-forming units per mL (pfu/mL) through the phage titration method (Smith and Scott, 1993; Bahadir et al., 2011).

### 2.6. ssDNA isolation from phages

After four rounds of biopanning, the final enriched MMP-2 specific clones were plated, and single pure plaques were isolated. Single-strand phage DNA was isolated from 30 peptide clones. Overnight *E. coli* TG1 cultures were refreshed (1/100 volume) in 5 mL nutrient broth. Phage plaques were collected from the plates using a pipet tip, added to *E. coli *TG1 cultures, and incubated for 4 h at 37 °C on a shaker. Infected cells were collected by centrifugation at 4000 rpm for 10 min. Supernatants (1 mL) were collected, and PEG8000/ 2.5 M NaCl solution were added. After incubation at room temperature for 10 min, the mixtures were centrifuged at 10,000 rpm for 10 min. The supernatants were discarded, and pellets were dissolved in 160 μL NaI and 400 μL ethanol. After incubation for 10 min, the mixtures were centrifuged at 10,000 rpm for 10 min. The pellets were washed with 70% ethanol. The samples were centrifuged again under the same conditions. Supernatants were discarded, and the sediments were dissolved in 30 μL sterile distilled water. The collected ssDNA were visualized through electrophoresis on 1% agarose gel (Tomley, 1993). 

### 2.7. Sequence analyses

To determine the peptide-encoding nucleotide sequence contained in each phage clone, sequence analysis was performed using the ssDNA of the phage as template. E. coli TG1 infected with phages were plated on medium with X-gal (5-bromo-4-chloro-3-indolyl-β-D-galactopyranoside)-isopropyl β-D-1-thiogalactopyranoside (IPTG) to obtain phage plaques. Thirty plaques were randomly selected. 

Sequencing reactions were performed as described in the Beckman Coulter Dye Terminator Cycle Sequencing (DTCS) protocol. The purified sequencing reactions were run on a CEQ 8000 DNA Sequencer (Beckman, Fullerton, CA, USA).

### 2.8. Zymogram

MMP-2 (15 ng) was incubated overnight with 2 ×10^11 ^pfu/mL phage clones that were enriched for binding against active MMP-2. After 18 h, each mixture was run on 7.5% SDS-PAGE with 20 mg/mL gelatin. After electrophoresis, the gel was washed twice at room temperature with 2.5% Triton-X 100 for 30 min. The gel was then kept overnight in zymogram retention solution (6.06 g Tris-HCl, 1.47 g CaCl_2_, and 2.92 g NaCl per liter). The next day, the gel was stained with Coomassie Blue R. The density of the bands were analyzed using the Bio-Rad Multi-Analyst program (Tajhya et al., 2017). The gel was scanned using a digital scanner, and MMP-2 activity was determined by measuring the peak area of each band. The peak percentage was normalized relative to the activity of MMP-2 (positive control) and wild-type M13 phage (negative control).

### 2.9. Peptide synthesis

Each MMP-2-specific peptide was synthesized (5 mg) and purified through high-performance liquid chromatography (HPLC) at 95% purity by Peptron Inc. (Daejeon, South Korea) for use in cell culture assays.

### 2.10. Cell culture assays

For cell culture study, human umbilical vein endothelial cells (HUVECs; C2519A) were purchased from Lonza, Basel, Switzerland. The cells were cultured in endothelial complete media, which is composed of endothelial basal media EBM-2 (CC-3156, Lonza) supplemented with 1% penicillin (10,000 units/mL)- streptomycin (10,000 units/mL) (Gibco, Cat No 15140122) and endothelial cell growth medium 2 (EGM-2) SingleQuots (CC-4176, Lonza, Switzerland) containing growth factors (insulin-like growth factor, human fibroblast growth factor, human epidermal growth factor, and vascular endothelial growth factor), and supplements (ascorbic acid, heparin, hydrocortisone, and fetal bovine serum). Cell passage was carried out three times per week, considering the cell doubling time (Erdag et al., 2011).

### 2.11. Cytotoxicity assays

A cytotoxicity assay based on impedance was performed using the xCELLigence real-time cell analyzer multiple plates (RTCA MP; Agilent Technologies, CA, USA). HUVECs were suspended in EBM-2 medium (CC-3156) and seeded at a density of 5 × 10^3^ cells per well into a disposable sterile 16-well E-plate of the xCELLigence RTCA MP. Various concentrations of MMPs (50, 100, 500, and 1000 μM) in EGM-2 complete medium were added to the wells. The cells were maintained in a humidified incubator at 37 °C with 5% CO_2_. The experiment was terminated at the end of the time period (72h), and the data were evaluated using the RTCA Software Pro software. 

### 2.12. Cell migration assays

Cell migration was investigated using a wound-healing assay. HUVEC cells were detached from the cell culture flask using 0.25% trypsin-EDTA and harvested when cell confluence was approximately 70%–80%. After confirming that cell viability was at least 90%, the cells were suspended in complete medium to a density of 1 × 10^5^ cells/mL. The cells were seeded (1 × 10^4^ cells/well) into 96-well plates (Greiner CellStar, USA), and incubated at 37 °C in a 5% CO_2 _humidified incubator for 24 h. After incubation, cell monolayers were gently scratched using AutoScratch (BioTek, USA) equipment to create repeatable wound areas. Cells were washed with medium to remove cell debris and treated with MMP-2 peptides (100 μM) in complete medium. EDTA (2 mM) was used as the positive control for MMP-2 inhibition. Cellular migration towards the wound area was captured and investigated every hour over a 24-h period using the 4× objective and the bright-field filter of the Cytation 5 (BioTek) cell imager. Wound areas were measured using the Gen5 software, and the relative wound area per time-point was calculated based on the wound at 0 h.

## 3. Results

### 3.1. Biopanning and phage amplification

In order to select 12-mer peptides that selectively bind active MMP-2, a 12-mer phage peptide library, which includes 4 × 10^10^ phages, was screened against APMA-activated MMP-9 to first exclude MMP-9-specific phages from further screening. Then, the remaining 12-mer peptides were screened against active MMP-2, and the peptides that bound active MMP-2 were recovered through elution (Figure 1).

**Figure 2:**
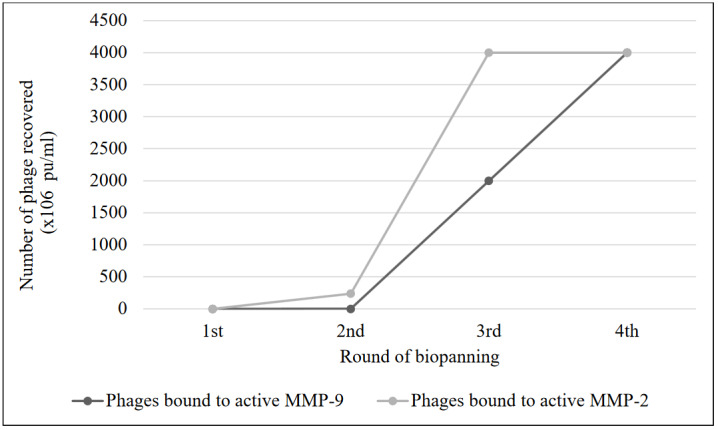
Recovery of matrix metalloproteinase (MMP)-9- and MMP-2-binding phages after each round of biopanning.

The biopanning cycle was repeated four times to determine MMP-2-specific peptides. After each cycle (except for the last cycle), the recovered and amplified phages were titrated (Bahadir et al., 2011, Smith and Scott, 1993) to ensure that the same number of phages was used for each biopanning cycle. Even with equal amounts of phage (1 × 10^12^ pfu/mL) used at the start of the selection cycles against MMP-2, we detected approximately 10^3^-fold increase in the number of phages recovered from the 3^rd^ cycle relative to number recovered in the 1st cycle (Figure 2). Biopanning was stopped as the number of phages obtained after the fourth round of biopanning did not increase (Figure 2). The quantities of phages bound to MMP-9 were also determined during the selection cycles. After the fourth round of biopanning, the number of phages bound to MMP-9 also increased.

### 3.2. Sequence and zymogram assay of phage plaques

**Figure 3:**
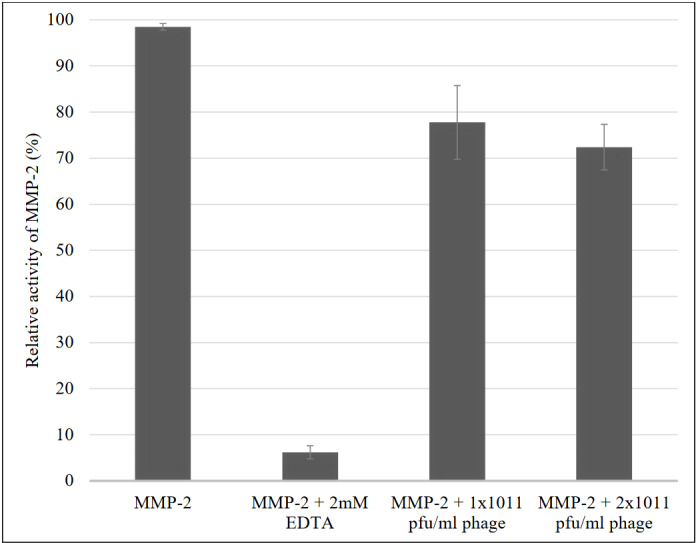
Effects of the M13 bacteriophage on matrix metalloproteinase (MMP)-2 activity.

Zymogram results were normalized relative to the activity of the wild M13 phage, which displayed approximately 25% inhibition of gelatinase activity on an SDS-PAGE gel with gelatin (Figure 3) (Lorenzl et al., 2003; Atkinson et al., 2004). 

**Figure 4:**
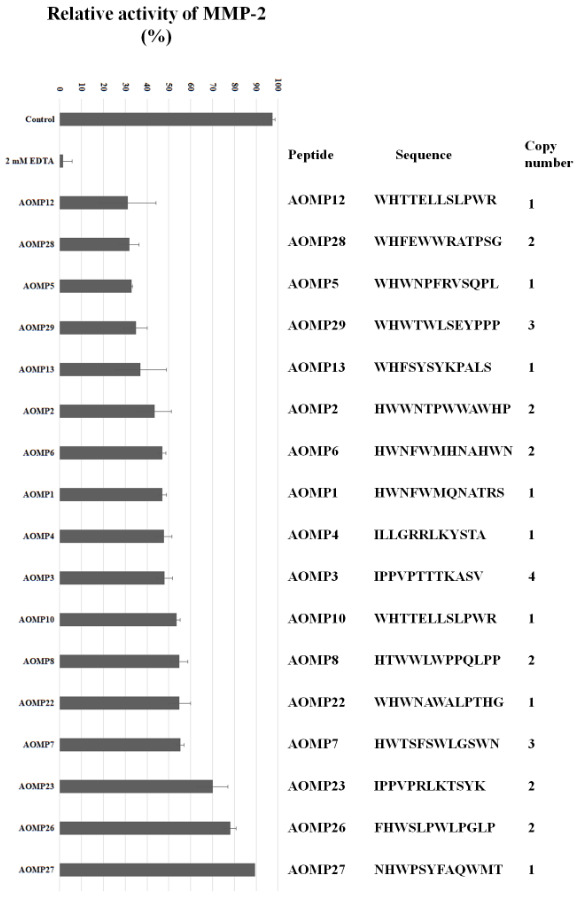
The different phage clones and their effects on matrix metalloproteinase (MMP)-2 activity. Amino acid sequences and copy numbers of the peptides are shown. The zymogram assay was used to measure inhibitory effects of the peptides on the gelatinase activity of MMP-2. EDTA (2 mM) was used as a control inhibitor of MMP-2 activity.

Thirty plaques were randomly selected for sequencing. The ssDNA from 30 phages were sequenced, and seventeen unique peptide sequences were identified. The sequences and copy numbers the peptides are shown in Figure 4. Based on the 17 unique peptide-encoding sequences, homologue sequence motifs, including WHW, HW, WH, or HWW, were observed in all but three peptides (AOMP3, AOMP4, and AOMP23).

The gelatinase inhibitory activities of all 17 phage clones were tested. Based on the gelatin degradation value obtained from the control sample (active-MMP-2), the percentage of gelatin degradation value was calculated for each phage clone. The peptides AOMP23, AOMP26, and AOMP27 showed lower MMP-2 inhibitory, with MMP-2 activity remaining over 60% (Figure 4). The best MMP-2 inhibitory peptides were AOMP5, AOMP 12, AOMP 28, AOMP 29, and AOMP 13, which reduced MMP-2 activity to under 40%. These preliminary findings indicate that our phage display selection of MMP-2 inhibitory peptides is a suitable screening strategy.

### 3.3. Cell culture assays

**Figure 5:**
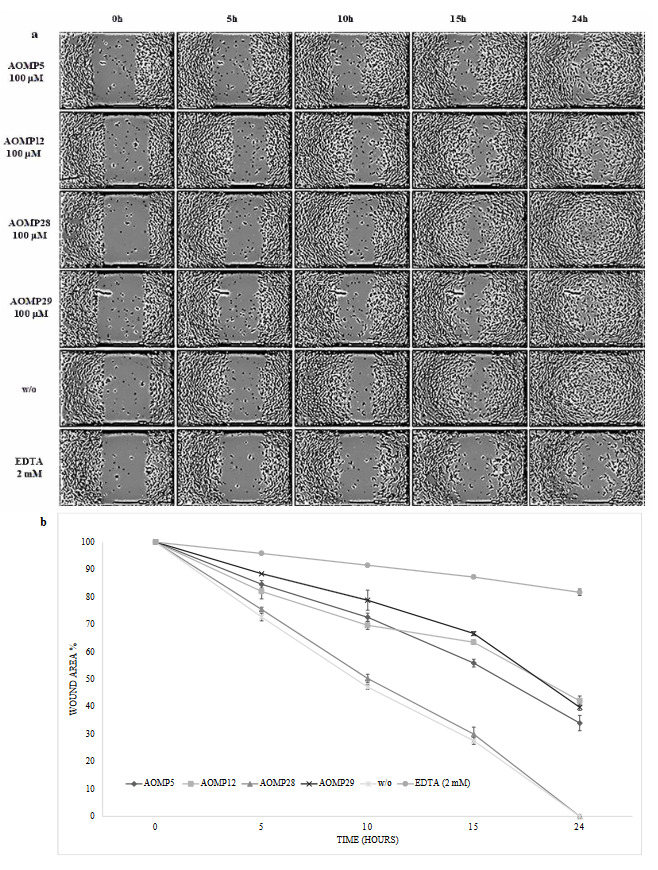
Effects of specific matrix metalloproteinase (MMP)-2 inhibitory peptides on endothelial wound healing. Human umbilical vein endothelial cells (HUVEC) monolayers were scratched using AutoScratch (BioTek) equipment to obtain repeatable and comparable wounds. Cells were treated with 100 μM MMP peptides. (a) HUVEC migration was monitored per hour under the 4× objective of the bright-field filter of Cytation 5 (BioTek) cell imager. (b) Relative wound areas at different time points were measured and calculated based on the wound at 0 h. Results shown are the means and SD from three independent experiments.

The top 4 peptides that inhibited MMP-2 activity (AOMP5, AOMP 12, AOMP 28, and AOMP 29) were synthesized for use in wound healing assays. First, the toxicity of the peptides on HUVECs were checked, and no toxicity was observed (data not shown). To determine the inhibitory effects of the peptides on HUVEC proliferation, a wound healing experiment was performed. Without peptides group was labeled as w/o. HUVEC monolayers were scratched, and the cells were treated with 100 μM of each peptide. After 24 h of incubation with 2 mM EDTA (as a control), no significant wound healing was observed, and 82% of the wound area was still uncovered (Figure 5a). Peptide AOMP28 did not inhibit wound healing. However, peptides AOMP29 and AOMP12 left a wound opening area of 42% and 40%, respectively.

## 4. Discussion

Gelatinases (MMP-2 and MMP-9) play essential roles in cancer progression and metastasis, as they can break down basal membranes. Therefore, the detection and inhibition of gelatinases is essential for cancer treatment.

Several studies have been conducted on the selection of MMP-2 and MMP-9 binding peptides from a peptide library. Koivunen et al. selected several peptides from a cyclic peptide library that bound both MMP-2 and MMP-9. They found that HWGF-containing cyclic peptides act as specific inhibitors of MMP-9 and MMP-2 (Koivunen et al., 1999). Trexler et al. screened a peptide library against the fibronexin 2 region of MMP-2, which interacts with the substrate (Trexler et al., 2003; Jani et al., 2005). Peptides obtained from these studies bound both gelatinases and displayed no selectivity for either MMP-2 or MMP-9.

In this study, we used a substractive biopanning strategy using a 12-mer peptide library for the selection of MMP-2-specific peptides. During the biopanning process, 10^3^-fold increase in phage production was observed from the first to the third biopanning steps, showing an enrichment against MMP-2-binding phages (Figure 2). Since phages which bind strongly to MMP-2 can also have a weakly affinity to MMP-9, these phages can also be amplified at each biopanning cycle. After the last round of panning, 30 phage plaques were randomly selected, and their peptide-encoding DNA sequences were determined. Sequencing identified 17 unique sequences and 13 duplicates of the sequences. The increase in the number of identical clones is proof of the enrichment of the library toward MMP-2. After sequencing, 14 peptide structures showed similar a.a. motifs (WHW, HW, WH, or HWW). The motifs we identified are similar to those that have been previously reported, indicating that peptides with high aromatic residue content are highly likely to bind MMP-2 (Koivunen et al., 1999; Trexler et al., 2003; Lu et al., 2012). A radiolabeled cyclic HWGF peptide, with similar motif to our peptides, showed promising results about the determination of gelatinase activity (Kuhnast et al., 2004). 

We also performed zymogram analyses using the peptides present on the phage surface. to test for their activity against MMP-2. The M13 bacteriophage without a 12-mer peptide on its surface did not inhibit MMP-2, so that the phages that displayed the 12-mer peptides could be used for the MMP-2 inhibition assay. After the zymogram tests, four of the 17 phage clones inhibited MPP-2 activity by 65%–70%. These four peptide structures were synthesized for further in vitro analyses. Peptide structures, all known to be non-toxic, were analyzed for wound healing, and we found that peptides AOMP29 and AOMP12 showed approximately 40% wound-healing inhibition (Figure 5b). Although EDTA, a chemical inhibitor, has twice the inhibition effect, the inhibition rate of the peptide structures is promising.

In conclusion, we have screened a 12-mer peptide library to identify peptides that block gelatinase A and isolated two new nanotechnological molecular tools specific for MMP-2. Cross-reactivity characterization with other MMP’s and subsequently the in vivo effects of anti MMP-2 peptides will be object of future work. 
